# Children’s Fruit and Vegetable Preferences Are Associated with Their Mothers’ and Fathers’ Preferences

**DOI:** 10.3390/foods10020261

**Published:** 2021-01-27

**Authors:** Kaisa Kähkönen, Mari Sandell, Anna Rönkä, Mika Hujo, Outi Nuutinen

**Affiliations:** 1 Department of Public Health and Clinical Nutrition, University of Eastern Finland, 70211 Kuopio, Finland; outi.nuutinen@uef.fi; 2 Department of Food and Nutrition, University of Helsinki, 00014 Helsinki, Finland; mari.sandell@helsinki.fi; 3 Functional Foods Forum, University of Turku, 20014 Turku, Finland; 4 Department of Education, University of Jyväskylä, 40014 Jyväskylä, Finland; anna.k.ronka@jyu.fi; 5 School of Computing, University of Eastern Finland, 70211 Kuopio, Finland; mika.hujo@uef.fi

**Keywords:** children, parental, fruit and vegetables, preference, food neophobia, eating competence

## Abstract

Children’s preference for fruit and vegetables must emerge during childhood. At children’s homes, mothers and fathers influence children’s developing food preferences with their own preferences and actions. The purpose of the study was to reveal the association parents have with their children’s fruit and vegetable preferences. The study was conducted in a sample of Finnish mothers and fathers of 3–5-year-old children. The participants were recruited, and questionnaires distributed through early childhood education and care centers in 2014 and 2015. The results showed considerable variance in the children’s preferences, and were more similar with their father’s, than their mother’s preference. There was an association between mother’s and children’s preference for “strong-tasting vegetables and berries“ (*p* = 0.005), “sweet-tasting fruit“ (*p* < 0.001) and “common vegetables“ (*p* = 0.037). Fathers preferences associated with children’s preferences for “strong-tasting vegetables and berries“ (*p* = 0.003). Food neophobia decreased children’s “strong-tasting vegetables and berries“ (*p* < 0.001) and “sweet-tasting fruit“ (*p* < 0.001) preferences. The father’s more relaxed attitude towards eating decreased children’s preferences for “strong-tasting vegetables and berries“ (*p* = 0.031) and “sweet-tasting fruit“ (*p* = 0.003). These findings indicate a need for more targeted strategies for increasing children’s preferences for fruit and vegetables and highlight the importance of taking both parents equally into account.

## 1. Introduction

Food preferences start taking shape during pregnancy and continue to develop throughout a person’s life [1]. These preferences, influenced by biological, social and environmental factors, are key elements of food choices and, consequently, diet quality [2,3]. Addressing fruit and vegetable preferences and consumption starting in early childhood is critical given that these dietary behaviors tend to persist into adulthood [4]. Furthermore, adequate consumption of fruit and vegetables is a key component of a health-promoting diet for chronic disease prevention [5] due to the low energy and high nutrient density, and high level of bioactive compounds [6] of these food items, which are crucial for cardiovascular disease [7] and obesity [8] prevention.

Fruit and vegetables are a diverse group of food items that are beneficial for health, and mainly consumed in too small quantities and infrequently. Fruit and vegetables are the food group most often rejected by children [9], but there is variation, depending on sensory properties, in rejection rates within the broad category of fruits and vegetables. Each fruit and vegetable type is unique in terms of its flavor, texture and appearance. While this makes the use of these items at meals and in cooking versatile, it also challenges children’s preference for them. For instance, the bitter flavor properties of species in the genus Brassica have been commonly identified as challenging for children to learn to like [10], and a high organic acid concentration [11] in berries decreases preference for them. By contrast, children tend to prefer fruit and vegetables with sweet flavor characteristics [12]. Indeed, the use of fruit and vegetables differ from item to item. Few studies have shown that the factors associated with consumption differ from one fruit or vegetable to another [13,14,15]. There is a lack of research concerning children’s and their parents’ fruit and vegetable preferences focused on specific items. In the present study, we hypothesized that there is interaction in the children’s and their parents’ preferences, and that preference is lowest for brassica vegetables and sour berries and highest for fruit.

Mothers’ food preferences start shaping children’s preferences in utero, and this continues to the postnatal period through breastfeeding [3]. As soon as parents start the complementary feeding of their child, they begin to influence their child’s food choices and the preferences that the child will eventually develop [16]. The association between parents’ and their children’s fruit and vegetable preferences is well-known [1,3]. Traditionally, mothers have played a greater role in food provision at home, but a recent increase in fathers’ involvement in feeding their children [17,18] raises interest in studying the family as a whole and also more specifically fathers’ influence on their children’s fruit and vegetable preferences. We hypothesized that observing the family as a whole would reveal that children’s preferences are more similar to those of their mothers than their fathers.

Children’s fruit and vegetable preferences are also directly related to their individual characteristics, such as genetics [19] and food neophobia [20]. Food neophobia is a characteristic closely related to food fussiness but specifically refers to the rejection of unfamiliar foods. Food neophobia is explained by both genetics and environmental factors [21]. Higher overall food neophobia has been consistently associated with lower consumption of vegetables [22] and fruit [23]. High parental food neophobia is especially crucial, as it is associated with lower overall diet quality, especially lower vegetable consumption [24] and may therefore limit the amount of fruit and vegetables offered to children in the home environment [25]. Our hypothesis is that food neophobic children, mothers and fathers tend to like fruit and vegetables less than their food neophile counterparts.

As the food-related decisions and behaviors during early childhood occur primarily in the home environment with parental presence, children are strongly influenced by their parents and their behavior [26]. There is little research available equally concerning both mothers’ and fathers’ influence on children’s fruit and vegetable preferences. Parents’ diet quality and fruit and vegetable consumption is associated with better economic and social resources. Higher socioeconomic status in parents increases children’s fruit and vegetable consumption [27,28,29]. As higher consumption at home is related to preferences, we hypothesized that fruit and vegetables are more preferred in families with more highly educated mothers and/or fathers.

Parents’ food-related behavior may be moderated by their own eating competence [30]. Eating competence is an attitudinal and behavioral concept that comprises four components: eating attitudes, food acceptance, internal regulation of eating, and management of the eating context. A higher eating competence is associated with higher diet quality and more frequent consumption of fruit and vegetables in adults [31] and school children [32,33]. Eating competent parents provide their children with fruit and vegetables more often at meals and as snacks [33,34]. So far, there is a lack of research on how parental eating competence components are associated with children’s fruit and vegetable preferences. Our final hypothesis is that parents’ eating competence components increase their children’s preference for fruits and vegetables.

In summary, children’s fruit and vegetable preferences have been related to food neophobia and their parents’ preferences. However, how these associations vary between fruit and vegetables with different sensory characteristics remains unclear. It seems probable that parents’ preferences and characteristics, such as food neophobia and eating competence, influence children’s preferences more with fruit and vegetables with sensory properties that are more challenging for children. Therefore, this study aimed to investigate whether children’s preferences for different kinds of fruit and vegetables are related to (a) parents’ preferences, (b) parents’ food neophobia, (c) children’s food neophobia, (d) parents’ education level and (e) parents’ eating competence components.

## 2. Materials and Methods

### 2.1. Participants

A total of 128 under-school-aged children (from 3 to 5 years old) and their parents (*n* = 113) participated in this study. The participants were recruited through ten groups of children at six early childhood education and care (ECEC) centers specialized in sensory-based food education in Western Finland and eight child groups at three standard ECEC centers in Eastern Finland. The parents of the 343 children in the ECEC centers were invited by a letter to participate in the study. Data collection took place in autumn 2014 and autumn 2015. Parental consent was obtained for 183 children (response rate of 53.4%).

The following inclusion criteria were applied: mothers and fathers of the 3–5-year-old children participating in the ECEC centers that had given their consent for the study, provided informed consent to participate. The following exclusion criteria for children were applied: food allergy, type 1 diabetes, coeliac disease. And the following exclusion criteria for the families were applied: single parent (*n* = 9), reconstituted family (*n* = 9), missing data in the questionnaires (*n* = 38). We received data from 145 children. 15 families had two participating children and the parents were instructed to answer separately for each child. At the ECEC centers, the ECEC instructors reminded parents about the study around two weeks after the questionnaires had been distributed.

Parents signed an informed consent form, and municipal directors of early childhood education and care and the managers of the participating ECEC centers also gave their consent. The University of Eastern Finland Committee on Research Ethics approved the study protocol (18 August 2014).

### 2.2. Questionnaires

The parents filled in the following separate questionnaires: (a) family background, (b) the fruit and vegetable preference questionnaire on their children’s and their own preferences, (c) the Food Neophobia Scale [35], the Child Food Neophobia Scale [36,37] and (d) the Satter Eating Competence Inventory, ecSI 2.0TM [30,38]. Family background and the home food environment information consisted of the child’s gender, the child’s age, parents’ age and parents’ level of education. Preference scores of 43 different fruit and vegetables were investigated with a 5-point hedonic scale (1 = I do/My child does not like at all, 5 = I like/My child likes a lot). The response option “I have/My child has not tasted the item” was included in the case of an unfamiliar food. These responses were marked as missing before statistical analyses.

Food neophobia was measured with the Food Neophobia Scale [35] and Child Food Neophobia Scale [36]. Parents rated their level of agreement with 10 statements on their children’s and their own behavior, for example, My child does not/I do not trust new foods, on a 7-point Likert scale (1 = strongly disagree, 7 = strongly agree). As half of the statements were worded in reverse relative to food neophobia, responses to these statements were reversed when calculating the score. Food neophobia scores were calculated as a sum of the responses, yielding a theoretical range of 10–70, higher scores indicating higher food neophobia.

Eating competence was measured using the preliminary Finnish translation [32] of ecSatter Eating Competence Inventory 2.0 (ecSI 2.0TM), which defines eating competence as comprised of four components: (1) eating attitudes; (2) food acceptance; (3) management of eating context; and (4) internal regulation of eating [39]. Eating attitudes (6 statements) comprise a positive, relaxed, and flexible interest in food and eating, and being responsively attuned to the inner and outer experiences relative to eating. Food acceptance (3 statements) means cognitive and behavioral processes and external influences on learning to accept and like a variety of foods, including new foods. The management of eating context (5 statements) prioritizes structure and meal planning as well as a permission to eat adequate amounts of the preferred food at predictable times. Internal regulation of eating (2 statements) denotes the experiential processes of hunger, appetite, and satiety. The ecSI 2.0TM comprises 16 Likert-scale statements, summed up to yield a total score and four subscale scores. The response options of the questionnaire were always, often, sometimes, rarely and never, and scored 3, 2, 1, 0 and 0, yielding a theoretical range of 0–48. A total score on the ecSI 2.0TM of at least 32 points indicates the presence of eating competence.

### 2.3. Statistics

The distribution of the data was verified nonparametric by using the Shapiro–Wilk test and visual inspection of histograms. As exceptions, children’s food neophobia, mothers age and mothers eating competence were normally distributed. Children’s fruit and vegetable preference scores were subjected to exploratory factor analysis. Fruit and vegetable items that more than 90% of the children tried were included in the analysis. To categorize these 28 fruit and vegetable items into fewer groups based on the preference scores, a factor analysis with varimax rotation was applied to component extraction. We ended up with the final number of factors on the basis of the following criteria: eigenvalue greater than 1, scree plots inspection, and meaningful factor content. For further analyses, we computed factor scores as means of preference scores of fruit and vegetables in each factor for children, mothers and fathers. Differences in the preference factor scores for children, mothers and fathers were tested with the Wilcoxon signed-rank test. The criterion for significance was set to be *p* < 0.05.

Associations with children’s fruit and vegetable preference factors were analyzed using linear mixed-effects models. This method was used due to statistical dependencies found among the children within the ECEC centers and child groups in the data. Dependencies were detected on two levels in this study. First, children socially influence one another in the ECEC groups, and second, the ECEC centers place the participants in specific geographic and infrastructural areas (e.g., local grocery stores’ size and selection). The linear mixed-effects model analysis method allowed us to take these dependencies into account. The model consists of a fixed and random part (described in detail [40]).

Identical procedures were carried out for all three models. Firstly, the children’s and parents’ fruit and vegetable preference factor scores, food neophobia, child gender and age, child’s ECEC centers’ implementation of sensory-based food education, parents’ education level and parents’ eating competence components were entered into the mixed model simultaneously as fixed factors and model diagnostics were performed. Statistically least significant fixed factors were removed from the model one at a time. The mixed model was re-run with all the remaining factors until only variables with close to significant (*p* < 0.1) associations remained in the final model. The fixed part of all three models included mothers’ preferences and child’s food neophobia. In addition, the strong-tasting vegetables and berries preference model included fathers’ preferences and fathers’ eating attitudes, the sweet-tasting fruit model included fathers’ preferences, fathers’ eating attitudes and fathers’ internal regulation, and the common vegetables model included child’s age and gender and mothers’ education level. When model residuals showed heteroscedasticity, variance functions [41] were included in the models to receive correct estimates for standard errors. Model checking was based on visual inspection of different residual plots and diagnostic plots on random effects.

The Wilcoxon signed rank test and factor analyses were completed with IBM SPSS Statistics 27.0 (IBM Corporation, Armonk, NY, USA). The mixed effects model analysis was performed using R version 4.0.3 and its nlme package [42].

## 3. Results

### 3.1. Subject Characteristics

Table 1 presents the characteristics of the children and their parents. Forty-one percent of the children were girls. The children’s ages ranged from 2.5 to 5.75. The parents’ ages ranged from 24 to 49 among mothers and from 25 to 54 among fathers. Roughly half of the mothers and one third of the fathers had a university degree. Children’s food neophobia was notably higher than that of the mothers’ and fathers’, which was at an equal level. Both mothers and fathers were defined on average as non-competent eaters (eating competence score ≤32 points). The total eating competence score ranged between 12 and 46 for mothers and 18 and 44 for fathers. In total, 46.0% and 32.7% of the mothers and fathers were eating competent (≥32 points).

### 3.2. Fruit and Vegetable Preferences

The children’s, mothers’ and fathers’ median preferences for fruit and vegetables are presented in Table 2. According to the parents, over 90% of the children had tried more than half (28/43) of the fruit and vegetable items. Among the parents themselves, only avocado, eggplant, kohlrabi and parsnip were tried by less than 90%. Children’s preference scores for most of the items were the lowest, mothers’ preferences scores were the highest and fathers’ preferences ranked in-between. Children’s median preference scores as compared with mothers and fathers were the lowest for 16 items (lingonberry, red/white currant, cloudberry, pineapple, grapefruit, onion, leek, eggplant, olives, kohlrabi, cauliflower and broccoli, swede and turnip, cabbages, champignon and other mushrooms) and fathers’ preference scores were the lowest as compared with children and mothers for 3 items (carrot, pear and other melons). Children’s scores were very high (median 5) for 12 items, mothers’ for 16 items and fathers’ for 11 items.

### 3.3. Factor Analysis of Children’s Preferences

In the factor analysis. we included children’s preference scores of 28 fruit and vegetable items that were tried by more than 90% of the children. Table 3 presents the components extracted from the factor analysis. For further analysis, three composite preference variables were extracted and labeled as strong-tasting vegetables and berries, sweet-tasting fruit, and common vegetables.

**Figure 1:**
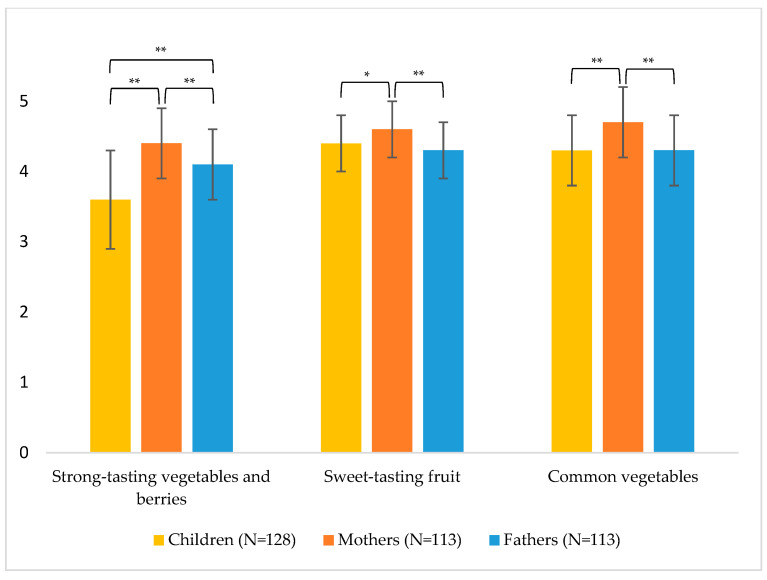
Results of the composite preference scores of 3–5-year-old children and their mothers and fathers (scale 1–5, 1 = I do/my child does not like this at all, 5 = I like/My child likes this a lot), mean and standard deviation. Significant differences shown * *p* < 0.01, ** *p* < 0.001 are based on the Wilcoxon signed rank test.

Children’s mean preference for strong-tasting vegetables and berries was significantly lower than that of mothers and fathers (*Z* = −8.88, *p* < 0.001 between children and mothers, *Z* = −6.41, *p* < 0.001 between children and fathers) (Figure 1.). Also, the preference for strong-tasting vegetables varied between mothers and fathers (*Z* = −4.86, *p* < 0.001). For sweet-tasting fruit and common vegetables, no significant differences were found between children and fathers, but mothers’ scores were significantly higher than those of children (*Z* = −2.86, *p* < 0.004 for sweet-tasting fruit and *Z* = −6.28, *p* < 0.001 for common vegetables) and fathers (*Z* = −4.10, *p* < 0.001 for sweet-tasting fruit and *Z* = −5.46, *p* < 0.001 for common vegetables).

### 3.4. Associations between Fruit and Vegetable Preference, Food Neophobia, Education Level and Eating Competence Components

The final linear mixed-effects models for the associations between the preference components related to fruit and vegetables in children and their parents, food neophobia, subject characteristics and eating competence components are presented in Table 4.

There was a positive association between mothers’ preference and children’s preference for strong-tasting vegetables and berries, sweet-tasting fruit and common vegetables, whereas fathers’ preference was significantly associated with their children’s preference for strong-tasting vegetables and berries, and also showed some, but not significant, association with the preference for sweet-tasting fruit.

Children’s food neophobia showed a negative association with children’s preferences in all three models. This association was strongest in the strong-tasting vegetables and berries model. No statistically significant association was found between preference components for children and mothers’ or fathers’ food neophobia.

Mothers’ higher education level decreased children’s preference for common vegetables. No association was found between preference components for children and fathers’ education level. Neither child’s age nor gender had significant associations with the fruit and vegetable preferences. However, there was a trend in the common vegetables model, indicating that the female gender and children’s older age might increase the preference.

Some significant associations were found between children’s fruit and vegetable preferences and parents’ eating competence components. Fathers’ attitudes towards eating decreased children’s preference for strong-tasting vegetables and berries and sweet-tasting fruit. On the contrary, fathers’ internal regulation increased children’s preference for sweet-tasting fruit. Of the eating competence components of mothers, only the positive association of food acceptance with the preference for common vegetables remained in the final models, but this association was not quite significant. Of the four eating competence components, the management of eating context and food acceptance showed no significant associations.

When exploring the statistical dependencies in the data with a mixed-effects model analysis, there seemed to be no effect from ECEC centers, but a slight variation was found between the child groups within the ECEC centers. Between these child groups in the centers, standard deviations were as follows: 0.25 for the strong-tasting vegetables and berries, 0.06 for sweet-tasting fruit and 0.12 for common vegetables.

## 4. Discussion

The present study explored the associations between children’s, their mothers and fathers’ fruit and vegetable preferences, food neophobia, education level and eating competence components. The study contributed to earlier literature, first by involving both parents of a child, in contrast to earlier studies only focusing on children and mothers. Furthermore, as a novelty, the current study examined parents’ eating competence, dividing it into four components and finding that fathers’ eating attitudes may limit their children’s fruit and vegetable preferences. Secondly, the study covered a wide variety of fruit and vegetables and identified from these three different groups: strong-tasting vegetables and berries, sweet-tasting fruit and common vegetables. Thirdly, in explaining children’s fruit and vegetable preferences, the study also took into account the ECEC environment and the children’s personal characteristics, thereby approaching children’s preferences in a more holistic way.

Children’s preferences varied considerably between the identified groups of fruit and vegetables, and both mothers’ and fathers’ preferences. Mothers’ positive influence, especially the effect of mothers’ preferences on their children’s preferences was visible in all fruit and vegetable groups, and an association related to fathers’ preferences was found in relation to strong-tasting vegetables and berries. The study reconfirms the well-established finding that children’s food neophobia plays a role in fruit and vegetable pleasantness. In this study, a negative association was found between mothers’ education level and children’s common vegetable preferences. Fathers’ more relaxed eating attitudes decreased children’s preferences for fruit and vegetables. These results highlight the importance of studying children’s families as whole, taking both parents equally into account.

### 4.1. Associations with Fruit and Vegetable Preferences

As was expected, preferences for fruit and vegetables varied and three groups were identified based on perceived pleasantness: strong-tasting vegetables and berries, sweet-tasting fruit and common vegetables. Children’s preference for sweet-tasting fruit and common vegetables was more similar with their fathers than mothers, whereas their preference for strong-tasting vegetables and berries differed from both parents. Our findings support the hypothesis that children’s preferences fluctuate, and brassica vegetables and berries that are strong in flavor are the least, and fruit that are often sweet-tasting most preferred by children. This finding was not as clearly consistent with the mothers and fathers.

As hypothesized, mothers’ preferences had greater influence on children’s fruit and vegetable preferences than did their fathers’. Preference for items among mothers increased the preference among children in all studied fruit and vegetables groups, and fathers had a particular positive influence on children’s preference for strong-tasting vegetables and berries. This result is in line with previous studies that have displayed the association between mothers and their children’s fruit [15] and vegetable [14] preferences.

Previous studies have shown that both genetic and environmental factors influence children’s fruit and vegetable preferences [43]. Also, it has been revealed that parents use different strategies for fruit and vegetables and more often negativity is present when eating vegetables as compared with fruit [44]. Mothers low preferences for vegetables might increase negativity in the eating-context and further reduce children’s preference for vegetables. Mothers low preference for vegetables reduces children’s exposure to them (14) and thereby decreases children’s chances to taste and learn to like them. In addition to repeated exposure, broadening the selection of vegetables and giving children a change to choose a vegetable they prefer can increase their consumption [44]

In the current study, children’s food neophobia associated with children’s preferences for strong-tasting vegetables and berries, sweet-tasting fruit and common vegetables, whereas parental food neophobia had no straight association with children’s preferences. This result regarding children is aligned with previous studies that have demonstrated a negative association between food neophobia and fruit and vegetable preferences [22,45]. Similarly, food neophobia is known to also be associated with lower fruit and vegetable preference in adults [46]. Although the results did not indicate a straightforward association, parental food neophobia might indirectly affect children’s preferences by lowering mothers’ and fathers’ own preferences and diminishing the quantity and frequency of fruit and vegetables offered to children. Previous studies have displayed that mothers who consume more vegetables are more likely to reoffer these to their children [47].

It is contrary to our hypothesis that mothers’ education level, an indicator of parental socio-economic status, was negatively linked to the preference for common vegetables among children. It could be that more highly educated mothers offer a broader selection of fruit and vegetables for their children, which challenges the children more and might take more time to learn to like. It must also be noted that more highly educated mothers might be more critical in reporting their children’s preferences. On the other hand, the result suggests mothers’ lower education level increases children’s preference for common vegetables. This is logical since the common vegetables included vegetables like cucumber and carrot that are both widely available and reasonably priced.

We hypothesized that the higher the parents eating competence components are, the higher are their children’s preferences for fruit and vegetables. Interestingly, we found that fathers’, but not mothers’, eating competence components were associated with the preference for strong-tasting vegetables and berries and sweet-tasting fruit among children. Fathers’ attitudes towards eating decreased children’s preferences for strong-tasting vegetables and berries and sweet-tasting fruit. Additionally, fathers’ internal regulation was positively associated with sweet-tasting fruit preference.

A new finding of this study was that a component of fathers’ eating competence, eating attitude, contributed negatively to children’s fruit and vegetables preferences. Although this was not the case with all the studied fruit and vegetables, it was apparent with strong-tasting vegetables and berries and with sweet-tasting fruit. A more positive eating attitude in the eating competence inventory implies a more positive, relaxed and flexible interest in food and eating [30]. It could be that fathers with such a relaxed eating attitude results in a greater influence of children on their food choices and less healthy food intake. This view is supported by previous studies that have indicated how fathers commonly let their children dictate their own food choices [48] and that fathers eating out with their children often opt for fast food restaurants [18]. It is more challenging to explain the result with internal regulation, and this might be due to coincidence.

We found no significant associations between children’s fruit and vegetable preferences and mothers’ eating competence components, although mothers’ eating competence has previously been shown to have a positive effect on mothers’ serving of fruit and vegetables already during their child’s infancy [49].

### 4.2. Limitations

Some limitations of this study need to be considered. First, children’s fruit and vegetable preferences were assessed by their parents (proxy-reporting). It is possible that when parents assess their children’s fruit and vegetable preferences, they mirror their personal preferences, which may lead to providing misinformation [50]. Moreover, the data may be affected by the parent who assesses their child’s eating, as parents may end up emulating their own habits [51]. Despite its limitations, a setting in which parents assess their children’s preferences is widely used and considered adequate when it is not possible to use the children themselves as informants. Second, fruit and vegetable preferences were assessed on paper and only the names of the items were given to support the respondents in forming a mental image of each item. It is likely that there were differences in the informants’ mental images related to the items, and as sensory properties, such as color, are known to affect perceived pleasantness, [52] this might have affected our results. Third, the sample was relatively small, and participants were recruited solely through ECEC centers and were limited to nuclear families. Excluding single-parent families may have limited the variation found in our data. Finally, although there were 15 families with two children participating, due to the limited number of participants, we ignored the issue of family affiliation and decided not to exclude half of these children.

## 5. Conclusions

The under-school-aged children’s fruit and vegetable preferences differed between studied items. Preferences for the food items were divided into three preference components: strong-tasting vegetables and berries, sweet-tasting fruit and common vegetables. The components were associated with both mothers’ and fathers’ preferences, children’s food neophobia, mothers’ education level and fathers’ eating attitudes. The associations varied for different preference components. These findings imply that there is a need for more targeted strategies for increasing children’s preference for fruit and vegetables. Future research should explore the role of fathers in preventing and promoting their children’s preferences for fruit and vegetables by way of questionnaire, interview and observational study designs.

## Figures and Tables

**Table 1:** Background information of the 3–5-year-old children (*n* = 128) and their mothers (*n* = 113) and fathers (*n* = 113).

Variable	Children	Mothers	Fathers
Age, years ^1^	4.5 ± 0.8	35.1 ± 5.1	37.2 ± 5.8
Sex			
Female ^2^	53 (41.4)		
Male ^2^	75 (58.6)		
Sensory-based food education at ECEC ^2^	72 (56.3)		
Level of education			
University ^2^		55 (48.7)	38 (33.6)
Lower than university ^2^		58 (51.3)	75 (66.4)
Food neophobia score ^1,3^	38.9 ± 11.7	25.0 ± 10.2	26.2 ± 13.0
Eating competence score ^1,4^		30.6 ± 5.5	29.8 ± 6.0
Eating attitude ^5,6^		13 (5–17)	13 (7–18)
Management of eating context ^5,7^		9 (0–15)	8 (0–14)
Food acceptance ^5,8^		5 (0–9)	5 (0–9)
Internal regulation ^5,9^		4 (1–6)	4 (0–6)

^1^ Mean ± standard deviation, ^2^ n (%), ^3^ theoretical range 10–70, ^4^ theoretical range 0–48, ^5^ median (min–max), ^6^ theoretical range 0–18, ^7^ theoretical range 0–15, ^8^ theoretical range 0–9, ^9^ theoretical range 0–6.

**Table 2:** Fruit and vegetable (FV) preferences (scale 1–5, 1 = I do/my child does not like this at all, 5 = I like/My child likes this a lot) of the 3–5-year-old children and their mothers and fathers.

FV	Children Who Have Tried A FV		Preference Score, 1–5		Mothers Who Have Tried A FV		Preference Score, 1–5		Fathers Who Have Tried A FV		Preference Score, 1–5	
	%	*n*	Median	Min-Max	%	*n*	Median	Min-Max	%	*n*	Median	Min-Max
Strawberry	100	128	5	2–5	100	110	5	4–5	100	106	5	1–5
Bilberry	100	128	5	2–5	100	110	5	4–5	100	106	5	3–5
Raspberry	100	128	5	1–5	100	110	5	4–5	100	106	5	3–5
Cucumber	100	128	5	3–5	100	110	5	2–5	100	106	5	1–5
Tomato	100	128	4	1–5	100	110	5	3–5	100	106	4	1–5
Lingonberry	99.2	127	3	1–5	100	110	4	1–5	100	106	4	2–5
Carrot	99.2	127	5	3–5	100	110	5	3–5	100	106	4	1–5
Salad	99.2	127	4	1–5	100	110	5	3–5	99.1	105	4	2–5
Pea, legume	99.2	127	4	1–5	100	110	4	2–5	99.1	105	4	2–5
Sweet corn	99.2	127	4	1–5	100	110	4	3–5	99.1	105	4	2–5
Banana	99.2	127	5	2–5	100	110	5	3–5	99.1	105	5	2–5
Apple	99.2	127	5	2–5	100	110	5	3–5	99.1	105	5	1–5
Pear	99.2	127	5	2–5	100	110	5	1–5	99.1	105	4	2–5
Orange	99.2	127	5	2–5	100	110	5	1–5	99.1	105	5	3–5
Grapes	99.2	127	5	2–5	99.1	109	5	3–5	98.1	104	5	2–5
Cherry tomato	99.2	127	5	1–5	100	110	5	2–5	100	106	5	1–5
Pineapple	99.2	127	4	1–5	100	110	5	3–5	99.1	105	5	1–5
Bell pepper	98.4	126	4	1–5	100	110	5	2–5	100	106	4	1–5
Other melons	98.4	126	5	1–5	99.1	109	5	1–5	99.1	105	4	2–5
Onion	98.4	126	3	1–5	100	110	4	1–5	100	106	4	1–5
Cauliflower, broccoli	97.7	125	3	1–5	100	110	4	2–5	98.1	104	4	1–5
Blackcurrant	97.7	125	4	1–5	100	110	4	2–5	100	106	4	1–5
Plum	95.3	122	4	2–5	99.1	109	4	3–5	99.1	105	4	1–5
Red/white currant	95.3	122	3	1–5	100	110	4	1–5	98.1	104	4	1–5
Apricot, nectarine, peach	93.0	119	4	1–5	100	110	4	2–5	99.1	105	4	2–5
Pickled cucumber	93.0	119	4	1–5	100	110	4	1–5	100	106	5	1–5
Gooseberry	93.0	119	4	1–5	100	110	4	2–5	98.1	104	4	2–5
Kiwi	92.2	118	4	1–5	99.1	109	4	1–5	98.1	104	4	1–5
Beetroot ^1^	85.9	110	4	1–5	99.1	109	4	1–5	99.1	105	4	1–5
Zucchini ^1^	84.4	108	3	1–5	100	110	4	1–5	99.1	105	3	1–5
Leek ^1^	84.4	108	2	1–5	100	110	4	1–5	98.1	104	4	1–5
Honeydew melon ^1^	83.6	107	4	1–5	100	110	4	1–5	96.2	102	4	1–5
Champignon ^1^	82.0	105	2	1–5	98.2	108	4	1–5	97.2	103	4	1–5
Cabbages ^1^	81.3	104	3	1–5	100	110	4	3–5	98.1	104	4	1–5
Swede, turnip ^1^	78.9	101	3	1–5	100	110	4	1–5	97.2	103	4	1–5
Other mushrooms ^1^	76.6	98	2	1–5	97.3	107	4	1–5	96.2	102	4	1–5
Cloudberry ^1^	71.9	92	3	1–5	99.1	109	4	1–5	99.1	105	4	2–5
Olives ^1^	69.5	89	2	1–5	99.1	109	3	1–5	99.1	105	3	1–5
Avocado ^1^	60.2	77	2	1–5	92.7	102	4	2–5	89.6	95	3	1–5
Parsnip ^1^	57.8	74	3	1–5	91.8	101	3	1–5	82.7	91	3	1–5
Grapefruit ^1^	48.4	62	2,5	1–5	96.4	106	4	1–5	98.1	104	4	1–5
Eggplant ^1^	42.2	54	2	1–5	88.2	97	3	1–5	84.0	89	3	1–5
Kohlrabi ^1^	35.2	45	2	1–5	73.6	81	3	1–5	78.3	83	3	1–5

^1^ The item was excluded from factor analysis as less than 90% of children had tasted it.

**Table 3:** The results of the factor analysis, rotated variable loadings of the extracted preference components for children (*n* = 128) (correlation coefficients). For simplicity, only coefficients above 0.400 are shown. The labels of new variables are in italics.

Fruit and Vegetables	Factor 1	Factor 2	Factor 3
*Strong-Tasting Vegetables and Berries*			
Red/white currant	0.766		
Blackcurrant	0.727		
Lingonberry	0.740		
Cauliflower, broccoli	0.661		
Pea, legume	0.632		
Cherry tomato	0.618		0.433
Bilberry	0.607	0.464	
Gooseberry	0.571	0.408	
Raspberry	0.552	0.493	
Tomato	0.517		0.514
Pickled cucumber	0.467		
Onion	0.455		
Sweet corn	0.442		
*Sweet-tasting fruit*			
Plum		0.776	
Banana		0.710	
Grapes		0.705	
Pear		0.683	0.432
Apple		0.622	0.496
Kiwi		0.558	
Strawberry	0.428	0.556	
Orange		0.549	
Other melons		0.527	
Apricot, nectarine, peach		0.511	
Pineapple		0.427	
*Common vegetables*			
Bell pepper			0.763
Salad			0.518
Cucumber			0.548
Carrot			0.504
Variance explained (%)	340.2	80.7	60.9

**Table 4:** The results of the linear mixed-effects model, fruit and vegetable preference components of 3–5-year-old children (*n* = 128) as dependent variables.

Dependent Variable	Estimate	Standard Error	*p*-Value
*Strong-Tasting Vegetables and Berries*			
Mother’s preference	0.40	0.14	0.005
Father’s preference	0.33	0.11	0.003
Child’s food neophobia	−0.02	0.005	<0.001
Father’s eating attitude	−0.05	0.02	0.031
*Sweet-tasting fruit*			
Mother’s preference	0.59	0.11	<0.001
Father’s preference	0.14	0.07	0.065
Child’s food neophobia	−0.01	0.003	<0.001
Father’s eating attitude	−0.05	0.02	0.003
Father’s internal regulation	0.11	0.03	0.003
*Common vegetables*			
Mother’s preference	0.27	0.13	0.037
Child’s food neophobia	−0.01	0.003	0.072
Mother’s higher education level	−0.26	0.09	0.003
Child’s age	0.09	0.05	0.089
Female gender	0.16	0.08	0.057
Mother’s food acceptance	0.05	0.02	0.058

## Data Availability

The data presented in this study are available on request from the corresponding author.
